# Severe Immune-Mediated Thrombotic Thrombocytopenic Purpura With High-Titer ADAMTS13 Inhibitors: A Case Report

**DOI:** 10.7759/cureus.112586

**Published:** 2026-07-13

**Authors:** Isabel V Rodrigues, Ana Pimenta de Castro, Elena Rios, Inês Barbosa, Paula Nogueira, Eva Lourenço

**Affiliations:** 1 Internal Medicine, Unidade Local de Saúde do Algarve - Hospital de Faro, Faro, PRT; 2 Intensive Care Unit, Unidade Local de Saúde do Algarve - Hospital de Faro, Faro, PRT

**Keywords:** acquired thrombotic thrombocytopenic purpura, adamts13 deficiency, adamts13 inhibitors, caplacizumab, factor b, factor h, immunosuppressive therapy, microangiopathic hemolytic anemia, rituximab, thrombocytopenia

## Abstract

Immune-mediated thrombotic thrombocytopenic purpura (iTTP) is a rare, life-threatening thrombotic microangiopathy caused by severe ADAMTS13 deficiency secondary to inhibitory autoantibodies. Prompt recognition and early initiation of treatment are essential to prevent irreversible organ damage and death. We report the case of a 46-year-old man who presented with constitutional symptoms, cutaneous purpura, severe thrombocytopenia, and microangiopathic hemolytic anemia. A PLASMIC score of 7 indicated a high probability of severe ADAMTS13 deficiency, which was confirmed by undetectable ADAMTS13 activity and high-titer inhibitory antibodies. The patient received therapeutic plasma exchange, corticosteroids, caplacizumab, and rituximab, resulting in rapid hematologic remission. An extensive diagnostic workup excluded alternative thrombotic microangiopathies. This case highlights the importance of early clinical recognition, systematic exclusion of alternative thrombotic microangiopathies, and prompt initiation of multimodal therapy in patients with suspected iTTP. It also illustrates the potential value of serial monitoring of ADAMTS13 activity and inhibitor titers as complementary biomarkers for assessing immunologic response and guiding therapeutic management.

## Introduction

Immune-mediated thrombotic thrombocytopenic purpura (iTTP) is a rare but life-threatening thrombotic microangiopathy characterized by severe thrombocytopenia, microangiopathic hemolytic anemia, and widespread microvascular thrombosis resulting from profound ADAMTS13 deficiency. In most cases, the deficiency is acquired and caused by circulating inhibitory autoantibodies directed against ADAMTS13, leading to the accumulation of ultra-large von Willebrand factor multimers, spontaneous platelet aggregation, and diffuse tissue ischemia. Congenital TTP accounts for only a small proportion of cases [1,2].

Historically, untreated iTTP carried a mortality exceeding 90%. The introduction of therapeutic plasma exchange dramatically improved survival, and more recent advances, including corticosteroids, rituximab, and caplacizumab, have further reduced mortality and accelerated hematologic recovery [3,4].

Nevertheless, relapse remains a significant clinical challenge, emphasizing the importance of close laboratory surveillance and long-term follow-up [4].

Because several thrombotic microangiopathies share overlapping clinical and laboratory features despite having different underlying mechanisms and treatment strategies, a systematic diagnostic approach is essential [5].

Current international guidelines recommend initiating treatment as soon as iTTP is clinically suspected, without waiting for confirmation of ADAMTS13 activity. Clinical prediction tools, particularly the PLASMIC score, are valuable for identifying patients at high risk of severe ADAMTS13 deficiency and supporting early therapeutic decisions while confirmatory testing is pending [6-10].

Here, we describe a patient with severe acquired iTTP presenting with high-titer anti-ADAMTS13 inhibitory antibodies, persistently undetectable ADAMTS13 activity, serial immunologic monitoring, and comprehensive complement evaluation, who achieved sustained hematologic remission following early multimodal treatment with therapeutic plasma exchange, corticosteroids, caplacizumab, and rituximab.

## Case presentation

A 46-year-old man with a history of psoriasis and ankylosing spondylitis presented to the Emergency Department with a one-week history of progressive asthenia, anorexia, and polyuria. He denied fever, vomiting, diarrhea, recent infections, or exposure to new medications.

**Figure 1:**
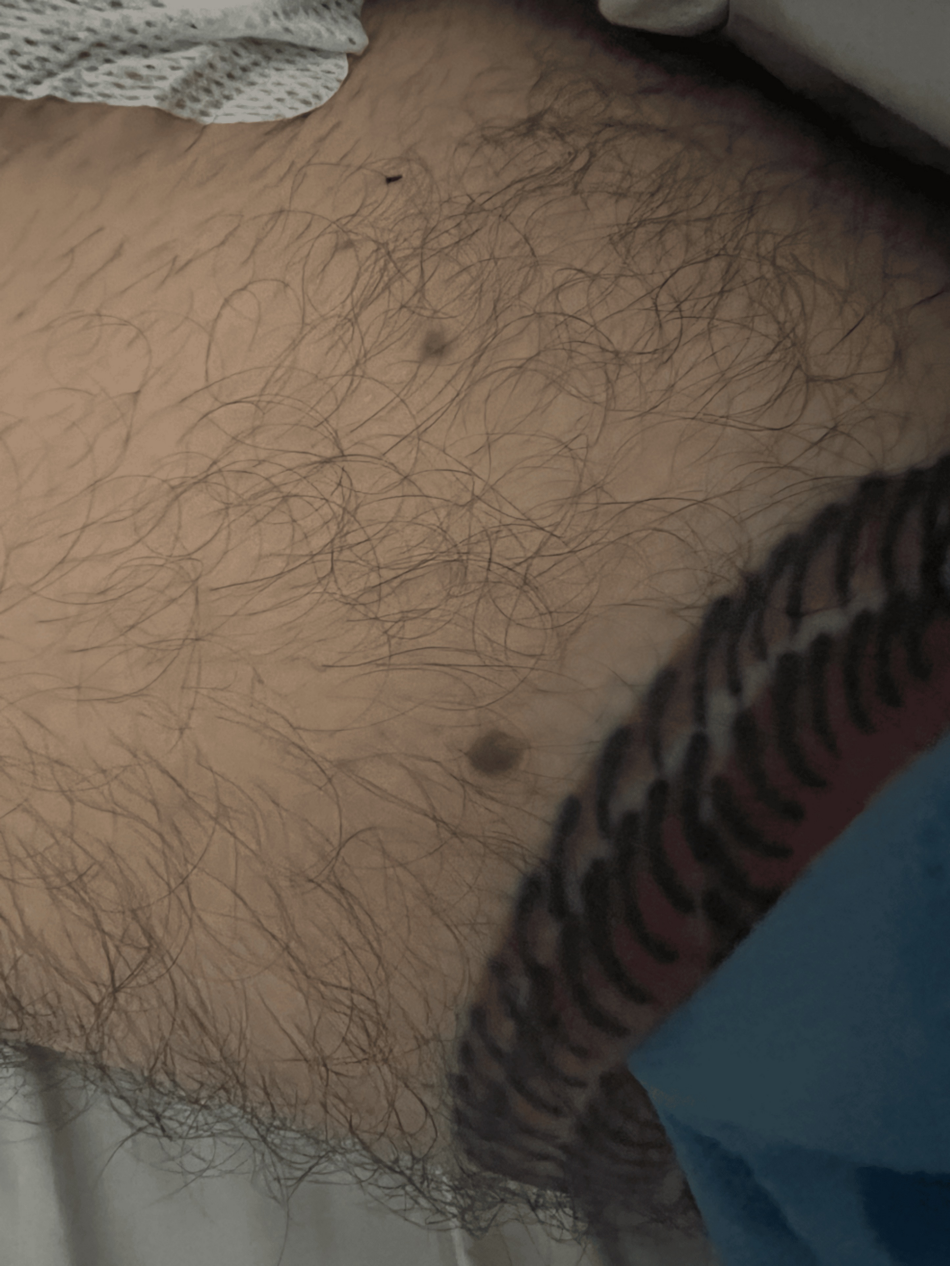
Cutaneous hemorrhagic manifestations of thrombotic thrombocytopenic purpura involving the left thigh. Clinical photographs of the left thigh showing multiple discrete, non-blanching erythematous-to-violaceous petechial and purpuric lesions of varying size. A larger, well-circumscribed purpuric macule is seen adjacent to smaller petechiae. No evidence of skin necrosis, ulceration, bullae, or secondary infection is observed. These findings are consistent with cutaneous hemorrhagic manifestations associated with severe thrombocytopenia and microvascular injury in thrombotic thrombocytopenic purpura.

**Figure 2:**
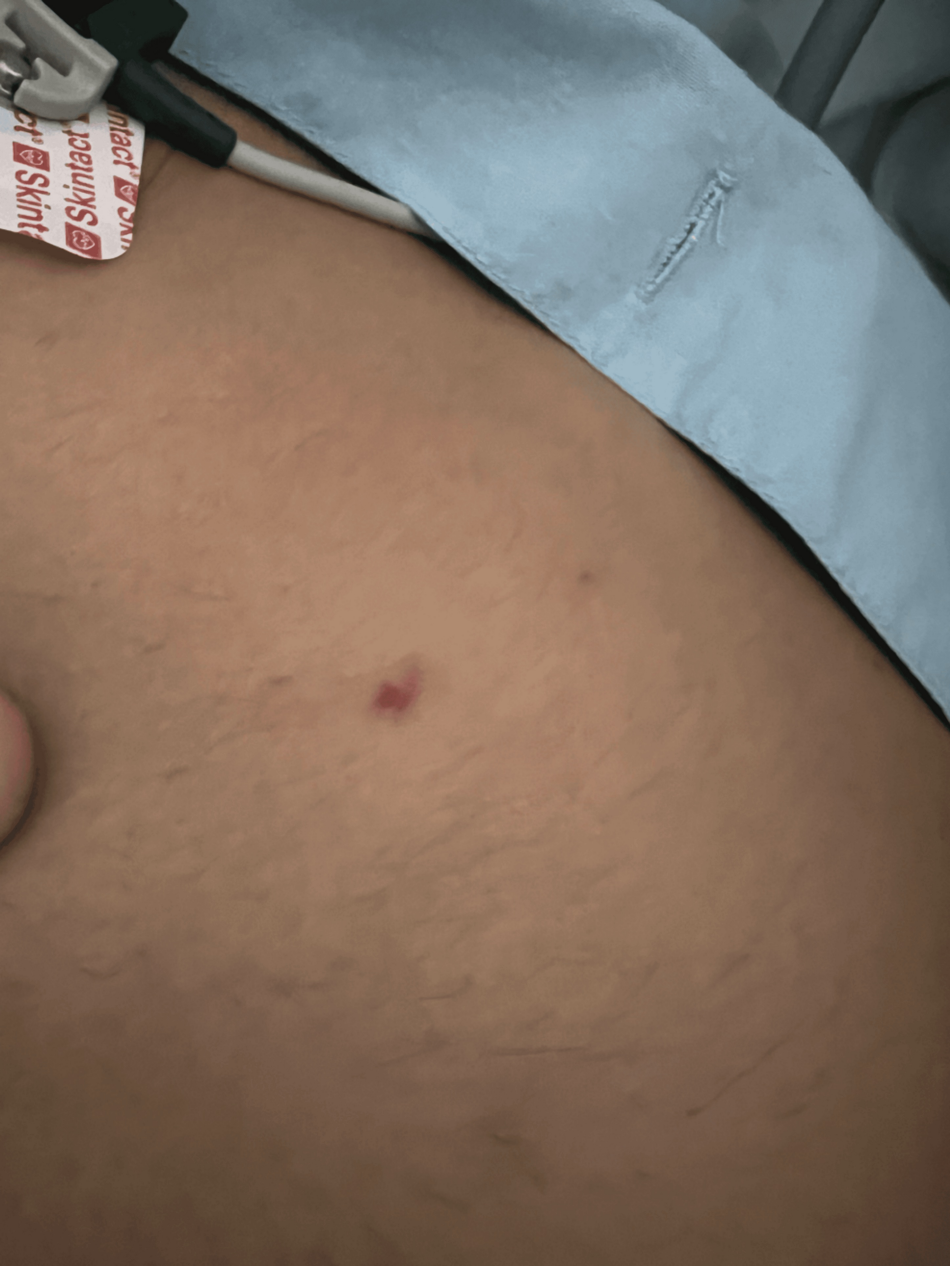
Isolated periumbilical purpuric lesion associated with thrombotic thrombocytopenic purpura. Clinical photograph demonstrating an isolated, non-blanching violaceous purpuric macule located in the left periumbilical region of the abdomen. The lesion is well-defined, non-palpable, and surrounded by normal-appearing skin, without evidence of necrosis, ulceration, or superimposed infection. The appearance is consistent with thrombocytopenia-related cutaneous bleeding in the setting of thrombotic microangiopathy secondary to thrombotic thrombocytopenic purpura.

Physical examination revealed two isolated cutaneous hemorrhagic lesions. Multiple discrete, non-blanching erythematous-to-violaceous petechial and purpuric lesions of varying size were present on the anterior aspect of the left thigh (Figure 1). In addition, a solitary, well-defined, non-palpable purpuric macule was observed in the left periumbilical region without evidence of necrosis, ulceration, or superimposed infection (Figure 2). Both lesions were consistent with thrombocytopenia-related cutaneous bleeding.

Initial laboratory investigations demonstrated severe microangiopathic hemolytic anemia (hemoglobin 7.3 g/dL), profound thrombocytopenia (22 × 10⁹/L), marked intravascular hemolysis (lactate dehydrogenase (LDH) >1000 U/L, haptoglobin <10 mg/dL, total bilirubin 2.55 mg/dL), and acute kidney injury (serum creatinine 1.8 mg/dL; baseline renal function unavailable), accompanied by proteinuria (2+) and microscopic hematuria (94 RBC/HPF (red blood cells per high-power field)) (Table 1). Peripheral blood smear demonstrated anisopoikilocytosis, polychromasia, keratocytes, pinched cells, and 6% schistocytes, consistent with active microangiopathic hemolysis. The calculated PLASMIC score was 7, indicating a high probability of severe ADAMTS13 deficiency [8].

**Table 1:** Initial laboratory investigations Baseline laboratory evaluation demonstrating severe microangiopathic hemolytic anemia, profound thrombocytopenia, biochemical evidence of intravascular hemolysis, and mild renal involvement, supporting the diagnosis of thrombotic microangiopathy. Abbreviations: RBC, red blood cell; MCV, mean corpuscular volume; RDW, red cell distribution width; ARC, absolute reticulocyte count; LDH, lactate dehydrogenase; RBC/HPF, red blood cells per high-power field.

Parameter	Result	Reference Range	Interpretation
Hemoglobin	7.3 g/dL	13.0–17.0 g/dL	Decreased
Hematocrit	21%	40–50%	Decreased
Red blood cell (RBC) count	2.58 ×10¹²/L	4.5–5.5 ×10¹²/L	Decreased
Mean corpuscular volume (MCV)	79.5 fL	83–101 fL	Decreased
Red cell distribution width (RDW)	16.6%	11.5–14.5%	Increased
Leukocyte count	10.8 ×10⁹/L	4–10 ×10⁹/L	Slightly increased
Platelet count	22 ×10⁹/L	150–400 ×10⁹/L	Markedly decreased
Reticulocytes	4.6%	0.5–2.5%	Increased
Absolute reticulocyte count (ARC)	117.6 ×10⁹/L	50–100 ×10⁹/L	Increased
Haptoglobin	<10 mg/dL	30–200 mg/dL	Decreased
Lactate dehydrogenase (LDH)	>1000 U/L	125–243 U/L	Increased
Creatinine	1.8 mg/dL	0.7–1.3 mg/dL	Increased
Total bilirubin	2.55 mg/dL	0.20–1.20 mg/dL	Increased
Direct bilirubin	1.00 mg/dL	<0.20 mg/dL	Increased
Proteinuria	2+	Negative	Present
Microscopic hematuria (RBC/HPF)	94 RBC/HPF	0–1 RBC/HPF	Present
Peripheral blood smear	Schistocytes (6%), anisopoikilocytosis, keratocytes, pinched cells, polychromasia	Normal peripheral blood smear	Abnormal

Baseline coagulation studies demonstrated normal prothrombin time, activated partial thromboplastin time, and fibrinogen concentration and the absence of lupus anticoagulant, findings arguing against disseminated intravascular coagulation (Table 2).

**Table 2:** Coagulation studies Baseline coagulation profile showing normal coagulation parameters and no evidence of disseminated intravascular coagulation or antiphospholipid syndrome. Abbreviations: PT, prothrombin time; INR, international normalized ratio; aPTT, activated partial thromboplastin time; DRVVT, dilute Russell's viper venom time.

Parameter	Result	Reference Range	Interpretation
Prothrombin time (PT)	12.4 s	9.4–13.0 s	Normal
International normalized ratio (INR)	1.1	0.8–1.2	Normal
Fibrinogen	3.33 g/L	1.8–4.8 g/L	Normal
Activated partial thromboplastin time (aPTT)	31.4 s	25.1–36.5 s	Normal
Normalized activated partial thromboplastin time ratio	1.01	<1.20	Normal
Dilute Russell's viper venom time (DRVVT)	Negative	Negative	Normal
Lupus anticoagulant	Negative	Negative	Normal

Given the broad differential diagnosis of thrombotic microangiopathy, an extensive diagnostic workup was undertaken. Real-time polymerase chain reaction analysis performed on a rectal swab was negative for pathogenic *Escherichia coli* virulence factors and Shiga toxin genes, excluding Shiga toxin-producing *E. coli* infection and hemolytic uremic syndrome (Table 3). Serologic testing for hepatitis B, hepatitis C, and human immunodeficiency virus showed no evidence of active infection, and blood cultures remained sterile. An interferon-gamma release assay was negative, excluding latent tuberculosis before rituximab administration (Table 3).

**Table 3:** Infectious and microbiological investigations Abbreviations: IGRA, interferon-gamma release assay; HCV, hepatitis C virus; HBsAg, hepatitis B surface antigen.

Investigation	Result	Reference Value	Interpretation
*E. coli* pathotype	Not detected	-	Negative
*eae* gene	Negative	-	Negative
*aggR* gene	Negative	-	Negative
*aaiC* gene	Negative	-	Negative
*ipaH* gene	Negative	-	Negative
Shiga toxin gene *stx1*	Negative	-	Negative
Shiga toxin gene *stx2*	Negative	-	Negative
Blood cultures (aerobic)	Negative	Negative	Normal
Blood cultures (anaerobic)	Negative	Negative	Normal
HBsAg	Negative	Negative	No active HBV infection
Anti-HCV antibodies	Negative	Negative	No HCV infection
HIV 1/2 Ag-Ab	Negative	Negative	No HIV infection
IGRA	Negative	Negative	No latent tuberculosis

Autoimmune evaluation, including antinuclear antibodies, anti-double-stranded DNA antibodies, ANCA (antineutrophil cytoplasmic antibodies), anti-glomerular basement membrane antibodies, antiphospholipid antibodies, and lupus anticoagulant, was negative. Complement C3 levels were within the normal range, whereas C4 levels were mildly elevated, arguing against autoimmune and complement-mediated thrombotic microangiopathies.

ADAMTS13 testing confirmed the diagnosis of iTTP. Initial assessment demonstrated complete ADAMTS13 deficiency (0%) associated with markedly elevated anti-ADAMTS13 inhibitor levels (80 U/mL), confirming the presence of pathogenic inhibitory autoantibodies. Serial measurements performed during treatment showed persistent severe ADAMTS13 deficiency with a progressive decline in inhibitor titers from 80 U/mL to 70 U/mL and subsequently to 40 U/mL, reflecting a gradual immunologic response (Table 4).

**Table 4:** Serial ADAMTS13 monitoring during treatment Serial ADAMTS13 activity and anti-ADAMTS13 inhibitor measurements demonstrating persistent severe ADAMTS13 deficiency with a progressive reduction in inhibitor titers during treatment, consistent with an evolving immunologic response.

Date	Timing Relative to Treatment	ADAMTS13 Activity (%)	Anti-ADAMTS13 Inhibitor (U/mL)	Interpretation
March 2026	Before initiation of therapeutic plasma exchange and immunosuppressive therapy	0	80	Severe acquired immune-mediated TTP
Day 7	During treatment (after initiation of therapeutic plasma exchange, corticosteroids, caplacizumab, and rituximab)	0	70	Persistent severe ADAMTS13 deficiency with declining inhibitor titer
Day 12	Follow-up during treatment	0	40	Persistent severe ADAMTS13 deficiency with continued decline in inhibitor titer

A diagnosis of acquired iTTP was established based on the clinical presentation, PLASMIC score, severe ADAMTS13 deficiency, and elevated anti-ADAMTS13 inhibitory antibodies. Treatment was initiated promptly with daily therapeutic plasma exchange and high-dose corticosteroids (prednisolone 1 mg/kg/day). Caplacizumab was added to inhibit von Willebrand factor-mediated platelet adhesion and microthrombus formation. Weekly rituximab therapy was initiated on Day 6 to suppress autoantibody production.

Serial laboratory monitoring throughout hospitalization demonstrated progressive hematologic recovery with normalization of haptoglobin, bilirubin, inflammatory markers, and renal function, together with a marked reduction in hemolytic activity, findings consistent with sustained hematologic remission following multimodal therapy (Table 5).

**Table 5:** Laboratory evolution after treatment Abbreviations: AST, aspartate aminotransferase; ALT, alanine aminotransferase; ALP, alkaline phosphatase; LDH, lactate dehydrogenase; CRP, C-reactive protein.

Parameter	Follow-Up Value	Reference Range	Interpretation
AST	17 U/L	5–34 U/L	Normal
ALT	28 U/L	<55 U/L	Normal
ALP	77 U/L	40–150 U/L	Normal
LDH	247 U/L	125–243 U/L	Near normalization
Creatinine	1.2 mg/dL	0.7–1.3 mg/dL	Normal
Total bilirubin	0.30 mg/dL	0.20–1.20 mg/dL	Normal
Direct bilirubin	0.15 mg/dL	<0.20 mg/dL	Normal
CRP	1 mg/L	<5 mg/L	Normal
Sodium	140 mmol/L	136–144 mmol/L	Normal
Potassium	3.6 mmol/L	3.3–5.1 mmol/L	Normal
Albumin	4.3 g/dL	3.5–5.0 g/dL	Normal

## Discussion

iTTP is a hematologic emergency associated with high mortality if treatment is delayed. Although the historical pentad of thrombocytopenia, microangiopathic hemolytic anemia, neurological abnormalities, renal dysfunction, and fever remains widely recognized, fewer than 10% of patients present with all five clinical features. Consequently, current practice emphasizes early recognition of thrombocytopenia and microangiopathic hemolytic anemia as sufficient grounds to suspect iTTP and initiate treatment while confirmatory ADAMTS13 testing is pending [7].

In the present case, the combination of profound thrombocytopenia, Coombs-negative microangiopathic hemolytic anemia, schistocytosis, markedly elevated lactate dehydrogenase, undetectable haptoglobin, and a PLASMIC score of 7 strongly suggested severe ADAMTS13 deficiency [8]. This allowed immediate initiation of therapeutic plasma exchange and corticosteroid therapy before laboratory confirmation, an approach consistent with current international recommendations for patients with suspected iTTP [9,10].

The introduction of plasma exchange has dramatically improved survival in patients with iTTP by removing circulating anti-ADAMTS13 autoantibodies while simultaneously replacing functional ADAMTS13 [9]. More recently, therapeutic strategies have evolved from plasma exchange alone to a multimodal approach combining plasma exchange, corticosteroids, caplacizumab, and rituximab, leading to further improvements in clinical outcomes [10].

The HERCULES trial demonstrated that early administration of caplacizumab significantly shortened the time to platelet count normalization, reduced disease exacerbations, and decreased refractory disease, establishing caplacizumab as a key component of first-line therapy in immune-mediated TTP [11]. In our patient, early incorporation of caplacizumab was associated with rapid hematologic recovery and sustained clinical remission.

Rituximab has become an important adjunctive therapy by suppressing anti-ADAMTS13 autoantibody production through B-cell depletion. Early administration during the acute episode has been associated with shorter disease duration, faster recovery of ADAMTS13 activity, and a lower risk of relapse during long-term follow-up [12]. The favorable clinical evolution observed in our patient is consistent with these findings and supports the use of early immunosuppressive therapy in combination with plasma exchange and caplacizumab.

Although current international guidelines are largely based on the recommendations issued by the International Society on Thrombosis and Haemostasis, additional national guidance has further refined the diagnostic and therapeutic approach to iTTP. The 2023 Japanese guidelines emphasize rapid diagnosis, early initiation of plasma exchange, prompt immunosuppressive therapy, and systematic monitoring of ADAMTS13 activity throughout the disease course, recommendations that closely parallel the management strategy adopted in the present case [13].

Recent comprehensive reviews have reinforced the concept that iTTP should be considered a chronic autoimmune disease characterized by recurrent immune dysregulation rather than a single acute episode. This perspective highlights the importance of continued clinical surveillance, laboratory monitoring, and individualized long-term follow-up even after complete hematologic remission has been achieved [14].

An additional noteworthy aspect of this case is the extensive diagnostic evaluation undertaken to exclude alternative thrombotic microangiopathies. Comprehensive microbiological, autoimmune, coagulation, and complement investigations excluded Shiga toxin-associated hemolytic uremic syndrome, autoimmune thrombotic microangiopathies, disseminated intravascular coagulation, and complement-mediated thrombotic microangiopathy. Such a systematic diagnostic approach is essential because several thrombotic microangiopathies may present with overlapping clinical and laboratory findings despite requiring substantially different therapeutic strategies [14,15].

In our patient, serial monitoring of ADAMTS13 activity and anti-ADAMTS13 inhibitor titers provided additional information beyond the initial diagnosis by documenting the progressive immunologic response to therapy. Recent evidence suggests that longitudinal ADAMTS13 assessment during clinical remission may help identify patients at increased risk of relapse and guide individualized follow-up strategies [16].

## Conclusions

iTTP is a life-threatening hematologic emergency in which early recognition and prompt initiation of therapy are essential to prevent irreversible organ damage and reduce mortality. This case highlights the importance of maintaining a high index of suspicion in patients presenting with thrombocytopenia and microangiopathic hemolytic anemia, even in the absence of the complete clinical pentad.

Serial ADAMTS13 monitoring may complement clinical follow-up and help identify patients at increased risk of relapse. Further studies are warranted to clarify its role in predicting disease recurrence and optimizing long-term management.
